# Loci That Control Nonlinear, Interdependent Responses to Combinations of Drought and Nitrogen Limitation

**DOI:** 10.1534/g3.118.200123

**Published:** 2018-03-01

**Authors:** Megan M. Chang, Danielle Allery Nail, Toni Kazic, Susan J. Simmons, Ann E. Stapleton

**Affiliations:** * Department of Biology and Marine Biology, University of North Carolina Wilmington, NC 28403; † Science Department, Green Hope High School, Cary, NC 27519; ‡ Department of Electrical Engineering and Computer Science; Plant Science Foundry; Interdisciplinary Plant Group; Informatics Institute; and Missouri Maize Center, University of Missouri, Columbia, MO, 65211; § Department of Mathematics and Statistics, University of North Carolina Wilmington, NC 28403

**Keywords:** quantitative trait locus, QTL, *Zea mays L*., complex phenotypes, phenotypic space, nonlinear response surfaces, mesh-based surface comparison

## Abstract

Crop improvement must accelerate to feed an increasing human population in the face of environmental changes. Including anticipated climatic changes with genetic architecture in breeding programs could better optimize improvement strategies. Combinations of drought and nitrogen limitation already occur world-wide. We therefore analyzed the genetic architecture underlying the response of *Zea mays* to combinations of water and nitrogen stresses. Recombinant inbreds were subjected to nine combinations of the two stresses using an optimized response surface design, and their growth was measured. Three-dimensional response surfaces were fit globally and to each polymorphic allele to determine which genetic markers were associated with different response surfaces. Three quantitative trait loci that produced nonlinear surfaces were mapped. To better understand the physiology of the response, we developed a model that reproduced the shapes of the surfaces, their most characteristic feature. The model contains two components that each combine the nitrogen and water inputs. The relative weighting of the two components and the inputs is governed by five parameters, and each QTL affects all five parameters.

We estimated the model’s parameter values for the experimental surfaces using a mesh of points that covered the surfaces’ most distinctive regions. Surfaces computed using these values reproduced the experimental surfaces well, as judged by three different criteria at the mesh points. The modeling and shape comparison techniques used here can be extended to other complex, high-dimensional, nonlinear phenotypes. We encourage the application of our findings and methods to experiments that mix crop protection measures, stresses, or both, on elite and landrace germplasm.

Crop improvement will need to accelerate in the coming decade, as the human population increases and the abiotic environment changes (Wheeler and von Braun 2013). Improving cultivars to be more resilient to stress is key. While breeding programs can increase the rate of genetic gain by using genotype prediction to shorten the breeding cycle time (Jonas and de Koning 2013), crop improvement depends on the immediate agricultural context, complicating selection schemes (Cooper *et al.* 2014). So far, cultivar improvement in maize has not significantly increased stress tolerance on a large scale (Lobell *et al.* 2014). But there is ample potential for improvement: in yield competitions, the maximum is typically one-third higher than the average (Tollenaar and Lee 2002).

Many breeding programs select germplasm in multiple environments, and model weather and soil inputs using multivariate methods or crop models. A crop model is an agronomic model of a crop’s productivity that incorporates genotypic, environmental, and management information into a system of equations (Cooper *et al.* 2016). Models used in predicting crop productivity via genomic selection and crop modeling make two sets of assumptions. The first set includes additivity and the choice of testing environments. To fit growth and to predict genotype-environment interactions, the current models typically assume an additive relationship among individual loci, the environmental and managerial contexts, and the phenotypes of interest. Using single genetic coefficients and a one-SNP-one-input relationship, the models partition the phenotypes’ variances into additive components that are each determined by a single QTL (Lamsal *et al.* 2016; Cooper *et al.* 2016). Thus, these models imply that any QTL candidate can be exchanged for another that exhibits an effect. Epistasis among the QTL is not considered, except as a last resort in interpreting the data (Huang and Mackay 2016). To predict the phenotypes of a line in a novel environment, current approaches attempt to infer performance from the same or genotypically related lines in a set of testing environments. These environments do not necessarily include the most predictive ones under climate change (Cairns *et al.* 2013). Further, the equations used to characterize environments can be nonlinear in the crop models, but the genotypes’ interactions with the environments are usually modeled linearly. These complications decrease prediction accuracy in many circumstances (for example, see (Cooper *et al.* 2016; Chenu *et al.* 2017)).

To understand the second set of assumptions, it helps to clarify several ideas about phenotypes. We define a complex phenotype as a function of at least three dimensions, of which at least one is an input, or independent, variable and at least one is an output, or dependent, observed variable. The set of dimensions can include measurements of different features of the phenotype, genotypic information, and environmental and cultivation conditions. The set of dimensions forms a point in an *n*-dimensional space, and each point corresponds to an experimental unit such as a plant, row, or plot. Each phenotypic point represents a particular instantiation of the phenotype. Together, the set of points that have the same definitions for each dimension comprise a phenotype. The points lie in a phenotypic space, and the surface they delimit is a phenotypic surface. (Throughout, we use the more general term “surface” to denote surfaces in any number of dimensions, including the “curves” traced by the successive positions of a single point in an *n*-dimensional space.) Because the points are high-dimensional, the phenotypes they denote are also surfaces, not scalars. We will refer to the phenotype defined by a group of phenotypic points as the phenotypic collection when needed to distinguish that from the common genetic usage of “phenotype”.

The second set of assumptions is that the phenotypic surface is determined by a mathematically linear function of the genotypes and the cultivation contexts. Each of the terms in an equation relating a phenotypic response to genotypes, contexts, and noises has a coefficient and a single term whose exponent is either 0 or 1. The phenotypic surface is planar when viewed in *n*-dimensional space: no terms bend the surface. The mathematical mappings among the phenotypic responses, the markers, and the contexts are one-to-one and onto. But the multiple stresses of field environments fundamentally change the mathematics of the analysis in its dimensionality, independence, linearity, and mappings. Each measured stress and observed trait are dimensions of the complex phenotype of crop performance and the equations that describe it. When the response of a single trait to a single stress is a two-dimensional function lying in a plane, many of the two-dimensional techniques familiar to geneticists are valid. Combinations of two or more stresses, or changes in more than one phenotypic dimension, push the phenotypic response function from the plane to surfaces in three or more dimensions, making the phenotype complex. Here, two-dimensional regression is uninformative, since infinitely many two-dimensional functions lie equally well on a three- or higher-dimensional surface. Moreover, the mappings can now be few-to-many or even many-to-many.

Many statistical methods assume that the input dimensions are independent of each other and have the same distribution (independent, identically distributed variates). In practice, both assumptions are often violated by real data. When two or more dimensions are related to linked mechanistic causes, their behaviors can jointly change in unexpected ways that are not apparent from covariance analysis. This interdependence of dimensions can occur both among the inputs and between inputs and outputs. Moreover, the distributions of values for each of the dimensions can be very different, even after rescaling, recentering, and other modest transformations. Finally, regression and many dimension reduction techniques assume that linear relationships exist between independent input dimensions and independent output dimensions. (Throughout, we use the term “linear” in its strict mathematical sense when referring to models. The statistical “linear” models we fitted included quadratic terms (see Equations 1 and 3), as statisticians use the term.) In at least some situations, these input-output relationships are not linear. Plotted in two or more dimensions, curvy or bumpy responses signal nonlinear relationships among the dimensions, and scatter beyond measurement and physiological noise can signal important missing dimensions or many-to-many mappings. To understand performance in the out-of-range field environments of the future, we will need a mechanistic view that incorporates climate prediction, an understanding of the genetic architecture and physiology of complex phenotypes, and analytical treatments that are appropriate to the data (Tardieu and Parent 2017; Chenu 2015; Cooper *et al.* 2014; Heslot *et al.* 2014). Genetic analysis of combined stress environments is a first step toward this mechanistic view.

There are examples of genetic dissection of abiotic stress combinations for heat and drought (Cairns *et al.* 2013). In the (Cairns *et al.* 2013) study, genotypes with good performance in drought or heat did not perform well when the stresses were combined (Hallauer *et al.* 2010). The poor predictive ability of single stresses for stress combinations illustrates the interdependence of single stress inputs in conferring the response. A controlled greenhouse-scale analysis of high UV stress combined with moderate drought also indicated that loci important for one stress did not have an important effect in the combined stress treatment; and that the combined stress effect level was less than additive, indicating a nonlinear protective interaction between the two stresses (Makumburage *et al.* 2013). This suggests that experiments should incorporate multiple levels of each input abiotic stress in order to detect loci that are important for the interactions between stresses.

Though analysis of the genetic control of the response to combined stresses is rare, we have more information about physiological responses for combinations within one or a few genotypes. For example, plant protection chemical mixtures show interaction effects (Dashevskaya *et al.* 2013), as do biotic/abiotic combinations (Prasch and Sonnewald 2014; Kissoudis *et al.* 2014; Suzuki *et al.* 2014). A common theme across all the types of combinations examined is that the effect of the stress interaction is not easily derived from single-effect responses. It is also clear that a single, often severe stress treatment does not predict the response at lower stress levels (Mittler 2006; Tardieu 2012; Zandalinas *et al.* 2017). Work on mixtures of toxins illustrates the classes of phenotypes one might see in response to combined stresses. Organismal responses to mixtures of drugs and chemical toxins are grouped into modes of action such as concentration addition, independent action, synergy, antagonism, dose-level, and dose-ratio (Jonker *et al.* 2005). The shape of the responses to the mixtures defines these different modes of interaction. In favorable cases, mechanistic inferences can be drawn from an analysis of the phenotypic responses to increasing levels of two abiotic stresses. Models of high-dimensional response surfaces can then be translated into network models (Lucas *et al.* 2011; Keurentjes *et al.* 2013; Reymond *et al.* 2003).

World-wide, one of the most important stress combinations in maize is co-occurring drought and nitrogen deficiency. Increased growth and yield in maize under drought and low nitrogen are genetically correlated; selection for one stress results in enhanced performance in the other stress environment (Weber *et al.* 2012). However, the correlation can vary by trait and by the specifics of the stress level and genotype used (Bennett *et al.* 1989; Sadras and Richards 2014). Typically, only a few levels of nitrogen and drought are applied; and factorial analyses are used instead of surface-fitting approaches that could compare equivalent stress intensities. The maize inbred lines B73 and Mo17 have different responses to drought and nitrogen. B73 exhibits top-fire and Mo17 barrenness under drought (A. Hallauer, personal communication), and their response to nitrogen differs by ≈25% (Balko and Russell 1980). These two inbreds are known to combine well as a hybrid (Hallauer *et al.* 2010), with the hybrid having very good performance under drought stress (A. Hallauer, personal communication). We infer that there are interactions between alleles of one or more genes in these parents that confer increased stress tolerance in the hybrid. If true, a population of offspring from these parents would generate a range of allele combinations from those inherited alleles, and would exhibit different responses to varying combinations of stresses. We also infer from stress combination experiments and toxicological data that sets of specific alleles should be able to control the sensitivity of growth to combinations of stresses, and thus shift the stress-tolerance system in maize among more-responsive and less-responsive states. Those states delimit parts of the phenotypic space that defines the set of high-dimensional, nonlinear stress response surfaces. The difficulties in predicting the effects of stress combinations from experiments using single stresses indicate physiological interactions among these dimensions.

Mapping the alleles that control these shifts in phenotypic space identifies the portions of the system controlling these responses. To better understand the mechanisms of field-relevant stress responses, the interaction between limited nitrogen and limited water in maize should be examined over a large range of levels of the stresses and in multiple genotypes with appropriate comparisons of the surfaces, rather than scalar summary statistics. In this paper, we map several alleles controlling overall responses to combined stresses, and identify the most parsimonious nonlinear producing function that describes their underlying mechanism. Identifying alleles, response surfaces, and models will be helpful in optimizing crop improvement strategies.

## Materials And Methods

### Seed Stocks

The *Zea mays* intermated recombinant line population (IBM94) derived from inbreds B73 and Mo17 (Lee *et al.* 2002) was provided by the Maize Co-op (http://maizecoop.cropsci.uiuc.edu/). Seed stocks were increased using standard nursery conditions at the North Carolina Central Agricultural Station, Clayton, NC. Seed lots were genotyped using eight simple sequence repeat markers; lines Mo066 and Mo062 failed genotyping quality control, and were thus removed from the data analysis. The B73 parent inbred was used for random checks across factor levels within the experiment.

### Experimental Design and Plant Growth

A face-centered cubic experimental design (Pukelsheim 2006) with five levels of drought and five levels of nitrogen was used to examine dose response surfaces for mixtures of the two stresses. The statistical program JMP v.6’s (SAS, Inc., Cary, NC, USA) experimental design module was used to compare design matrices and to generate the face-centered cubic sample points (see Figure S7). This experimental design has more biological replicates in the center portions of the response surface to enable better fit of nonlinear functions. We used an unbalanced design to increase the power for detecting the genotype-stress combination QTL, as QTL analysis was the primary goal of this experiment. In our face-centered cubic experimental design, the maximum sample size was either n=4 or n=8. The seeds of each of the 89 IBM RILS and parental Mo17 and B73 inbreds were randomly assigned within the cubic centered face design stress levels; replication existed within these levels of the experimental design. Pots in water levels were grouped to reduce human error when administering the water to the plants. Therefore, the only variation that would confound the experiment would be spatial variation within the greenhouse, *i.e.*, if there was fluctuation or variation between parts of the greenhouse table within a water level that affected the estimation of the genotype-environment interaction. We considered adding spatial modeling of water blocks to our analysis but the additional complexity could not be justified, since differences in temperature or light across the greenhouse tables were not detected. The experiment was conducted in the Cape Fear Community College horticulture greenhouse (GPS coordinates Lat: N 34° 19′ 24${}^{\prime \prime }$ (34.324°) Lon: W 77° 52′ 45${}^{\prime \prime }$ (-77.879°), weather station KNCCASTL2) from May–July 2011. Greenhouse maximum temperature was set to 38°C.

Slow-release fertilizer was custom-mixed by Coor Farm Supply, Smithfield, NC, with clay pellets containing standard trace minerals, 15% potassium, 15% phosphate, and nitrogen levels of 0, 2.5, 7.5, 12.5, and 15% fertilizer treatment level. 6.36 kg of fertilizer pellets were mixed with a 0.08 m${}^{3}$ bag of MetroMix360 potting mix (SunGro, Vancouver, BC, CA). Deep plant pots (MT38, 0.9 l, Stuewe and Sons, Tangent, OR, USA) were filled with soil-fertilizer mix. Random soil-filled pots were weighed, with an average weight of 350 g per pot. Seeds were planted 1 cm below the soil surface. A random number was generated for each plant pot within each water level using SAS v9.2 (SAS Inc. Cary, NC, USA). The plant pots were sorted by random number within each water level, so that neighboring plants were of randomly chosen genotypes and nitrogen levels. Water evaporation in plant pots containing B73 checks in the greenhouse was examined May 20–24; the average difference in weight between fully wet and dry pots over 24 hr was 170 g. Drought was applied to experimental groups using this average, with drought levels of 8, 20, 50, 80, and 92% of full weight (13 ml water, 34 ml water, 85 ml water, 136 ml water, and 156 ml water applied *per* day *per* pot). Selective watering in different amounts was applied from 20–30 days after planting, beginning when the check plants were at the four-leaf growth stage. Soil water potential was measured with a conductivity meter (EC-5, Decagon Devices, Pullman, WA, USA); the selective watering was stopped when the B73 check 8%-weight plant pots had an average water potential of 2%. All pots were watered fully for five days after drought treatment.

### Trait Data Collection

Each plant pot was photographed against a 1 cm grid background 14 days after planting, before selective watering. Plants were re-photographed using the same setup and focal length 35 days after planting, after recovery from selective watering.

Plant photographs were measured using ImageJ (Schneider *et al.* 2012), with the internal centimeter ruler in each image used to calibrate the pixel lengths for each measurement session. Each person analyzing the images practiced on a calibration image set until his or her accuracy was greater than 95%. All plant images are available upon request. The complete trait data file is included as Supplemental Data Files 2 and 3.

### Parental Inbred Analysis

The initial plant height was subtracted from the final height to generate $Z_{ijk},$ the growth variable difference_in_height. Measured initial, final, and difference in plant heights for each parental inbred for each stress treatment were fit with a full factorial model (see Equation 1) using JMPv11 (SAS Inc., Cary, NC).

### Mixture Surface Parameter Fits

For the check B73 inbred with adequate data points, the height difference data were analyzed by the procedure of Jonker *et al.* (2005) using Excel Mixtox analysis tools provided by C. Svendsen. The Mo17 data had too few points to generate a fit. The first step in the Mixtox analysis procedure was to fit a single dose response relationship to the height difference data using the log-logistic two-dimensional surface as a dose response model to determine the separate, single parameter effects of water and nitrogen. To fit the surface to the water level, the data were filtered to only include points with the lowest level of nitrogen that had a variation in the water level — in this case, the nitrogen level was held constant at 2.5% in order to analyze the single parameter effect of water. Similarly, the surface was fit for single-parameter nitrogen by holding the water level constant at 20%. These fitted surfaces are the empirical analogs of the discrete partial differentials of the response surface with respect to water or nitrogen, constrained to lie on the planes where nitrogen =2.5% and water =20%, respectively. The fits of the log-logistic surfaces were further refined using the Solver add-in in Excel to minimize the sum of squares (SS) between the actual data points and the predicted model values. The second step in the analysis was to fit the Mixtox mixture dose-ratio and dose-level reference models and the deviation models to the data. In order to optimize use of the solver, which was set up to use one measurement rather than replicates, and was not optimized for a face-centered cubic design, an average was taken for each of the treatment combinations in the larger data set.

### QTL Analysis

To determine which markers are responsible for creating significantly different dose response surfaces, we fit the response data for each marker, then compared these to the surface fit to all the markers from all lines using an *F*-test (see Supplemental Materials 6). Since our experimental design was optimized to detect interactions among markers and stresses, we fit the data to a quadratic function; and we focused on smoothed surfaces to incorporate all the information across levels efficiently. As seeds germinate at different times, all the recombinant inbred analyses were conducted on trait measurements adjusted for initial plant size. This approach fits the data for $Z_{ijk},$ the difference in height, using Equation 1:

$$\begin{array}{l} Z_{ijk}=\mu +\alpha_{i}\;+\beta_{1}x_{w,j}+\beta_{2}x_{n,k}\\ \;+\beta_{3}x_{w,j}^{2}+\beta_{4}x_{n,k}^{2}+\beta_{5}x_{w,j}\;x_{n,k}+\epsilon_{ijk}, \end{array}$$ (1)

where $Z_{ijk}$ is the difference in height for line *i* before and after the combined water $x_{w},$ and nitrogen, $x_{n},$ stresses; *μ* is the mean of the height differences before and after stresses; $\alpha_{i}$ is a random effect due to line *i*; $x_{w,j}$ is a covariate for the $j^{\text{th}}$ amount of water; $x_{n,k}$ is a covariate for the $k^{\text{th}}$ amount of nitrogen; and $x_{w,j}\;x_{n,k}$ is the interaction term between j=1,2,…,J for *J* amounts of water and k=1,2,…,K for *K* amounts of nitrogen. $\beta_{\cdot }$ are the regression coefficients, and *ϵ* is the residual error in the fit to the data.

To fit the data to this equation and detect QTL, we first fit an all-inclusive model that included all the markers from all the lines (Motulsky and Christopoulos 2004). Then for each marker, data from all the lines and all combinations of water and nitrogen were divided into two groups according to whether the genotype of the marker was B73 or Mo17. The SAS procedure PROC MIXED was used to model each surface. Due to non-random relatedness between recombinant inbred lines, we incorporated kinship matrix information into the analysis, as recommended by (Malosetti *et al.* 2011). Kinship matrices were calculated using the SPAGEDI method (Hardy and Vekemans 2002) within the TASSEL v3 program (Bradbury *et al.* 2007).

The sums of squares for the individual marker models were compared to the sums of squares of the all-inclusive model via an F-statistic. The resulting raw *P*-values from the analysis were adjusted using the approach described by Makumburage and Stapleton (2011), by grouping correlated adjacent marker *P* values with the Simes function in SAS (PSMOOTH). SAS data steps were used to scan the Simes-adjusted *P* values for groups of significant markers adjacent along the chromosome. A false discovery rate of 0.05 and a Sidak adjustment of 0.05 were each separately used to adjust for multiple testing. Raw and adjusted *P*-values, marker data for each mapping line, and SAS code for surface fits and *P*-value adjustment are provided in the Supplemental Data and Methods files (1, 4, and 5).

### Response Surfaces of the Markers

We generated the phenotypic response surfaces of the lines using the parameters obtained by linear regression (Table 1) and Equation 1. These surfaces were plotted in three-dimensional Cartesian space using the R package rgl, recentering the intervals for water and nitrogen (Adler *et al.* 2017–present). Plotting details and code are provided in Supplemental Methods File 11. For each marker, two response surfaces were plotted for the B73 and Mo17 alleles in the QTL region.

**Table 1:** *Regression Coefficients and Constant Terms for the Experimental Data Regressed to Equation 1*. The regression coefficients, $\beta_{\cdot },$ are ordered by the degree of the applied stresses. Thus, $\beta_{1},$ $x_{w};$ $\beta_{2},$ $x_{n};$ $\beta_{3},$ $x_{w^{2}};$ $\beta_{4},$ $x_{n^{2}};$ and $\beta_{5},$ $x_{w,n}.$ The columns are ordered to highlight the elliptical paraboloid ($\beta_{3}x_{w,j}^{2}+\beta_{4}x_{n,k}^{2}$), hyperbolic paraboloid ($\beta_{5}x_{w,j}\;x_{n,k}$), and plane ($\beta_{1}x_{w,j}+\beta_{2}x_{n,k}$), components of Equation 1 in that order. The lumped constant ℓ incorporates all the constant terms in the equation, $\ell =\mu +\alpha_{i,0}+\epsilon_{i,w,n}.$

*marker*	$\beta_{3}$	$\beta_{4}$	$\beta_{5}$	$\beta_{1}$	$\beta_{2}$	ℓ
B73	−0.006042	−0.188712	0.025567	0.229554	0.997363	32.034927
QTL1-B73	−0.004447	−0.110527	0.007851	0.251079	0.542153	29.572431
QTL2-Mo17	−0.005370	−0.105151	0.007068	0.239113	0.497335	30.499056
QTL3-Mo17	−0.005307	−0.116559	0.005924	0.249475	0.525478	31.324343
QTL1-Mo17	−0.003192	−0.049303	0.004224	0.215691	0.317815	26.144178
QTL2-B73	−0.002367	−0.068065	0.006244	0.237807	0.418609	25.779661
QTL3-B73	−0.002917	−0.063999	0.008152	0.228592	0.411333	25.720664
Mo17	0.000814	−0.001170	−0.006244	−0.009502	0.063104	0.266425

### Annotation of QTL Loci

QTeller (http://www.qteller.com/) was used to assemble a list of maize genes in the three chromosomal regions containing QTL that changed the difference in height $Z_{ijk},$ and the gene IDs were placed into AGRIGO (Du *et al.* 2010) for annotation analysis. All GO annotations within each QTL were used to create scaled semantic-similarity plots through the Revigo interface (Supek *et al.* 2011).

### Mathematical Model of the Phenotype

The most distinctive and robust feature of the plant height phenotype is the shapes of the response surfaces. We sought a function that was simpler than Equation 1, that would reproduce the shapes of the experimental phenotypes, and that would not assume the hypothesized interactions. The simplest such producing function is the sum of two components, an elliptical paraboloid and a plane, shown in Equation 2.

$$z=\underset{\text{elliptical}\;\text{paraboloid}}{\underset{︸}{c\left(ax_{w}^{2}+bx_{n}^{2}\right)}}+\underset{\text{plane}}{\underset{︸}{dx_{w}+ex_{n}}},$$ (2)

*z* is the difference in height; $x_{w},$ water; $x_{n},$ nitrogen. a,b, and c are parameters governing the paraboloid’s shape and orientation (*c*) and its weighting of input water (*a*) and nitrogen (*b*). d and e tilt the plane along the water and nitrogen axes, respectively. To confirm the model’s correctness, we tested many different candidate components by analysis and substitution, the importance of each term by deletion, and the effects of the parameters by simulation. Using R, the function was evaluated over the recentered water and nitrogen intervals [−42,42] and [−7.5,7.5] with a step size of 0.5, and surfaces were plotted in a standard orientation (Supplemental Files 12 and 16). Package viridis was used to color these and subsequent plots, since it produces color maps that are less problematic for those with color blindness (Garnier *et al.* 2017–present).

### Estimation of Model Parameters

We estimated the parameters of the producing function (Equation 2) for each allele’s surface using standard linear regression, analogous to the fit of Equation 1 but omitting the constant. We set the value of *c* to {−1,1} for the non-Mo17 and Mo17 shaped experimental surfaces, respectively, and estimated the values of (a,b,d,e) for

$$Z=c\left(aX_{w}^{2}+bX_{n}^{2}\right)+dX_{w}+eX_{n},$$ (3)

where Z is the vector of empirical heights at a mesh of points at coordinates $\left(X_{w},X_{n}\right).$ The mesh points were chosen to emphasize surface regions that had the most empirical data and were most comparable among the surfaces. The points lie at the intersections of a set of contours at fixed *z* values and a set of rays defined with respect to a local axis. For the non-Mo17 surfaces, the local axis was defined as the apparent major axis of the distorted ellipsoid. For Mo17, the local axis was defined as midway between the asymptotes of the trough. The rays extended from the surfaces’ peaks at fixed angles relative to the local axes. We call these the “absolute mesh points” to distinguish them from the relative mesh points of the next section.

We used the linear solvers Solve and lsei in the R package limSolve (Soetaert *et al.* 2009; Van den Meersche *et al.* 2009). Both gave identical parameter values. We report those obtained by lsei in Table 3, since that algorithm also computes a scalar error measure cumulated over the surface, the square root of the least squares error fit. We repeated these computations using the relative mesh points described in the next section. Those fits were considerably worse except for Mo17, as judged by the scalar error. Code for these computations is in File S15. The resulting surfaces were projected into the $\left(x_{w},x_{n}\right)$ plane and plotted using the image function of package graphics (R Development Core Team *et al.* 2017–present).

### Comparison of Surfaces’ Shapes

We assessed how well the surfaces generated by the model using the fitted parameters reproduced the intrinsic shapes of the experimental surfaces. For all possible pairs of experimental and simulated surfaces, we scored differences in signed Euclidean distance, *ρ*; relative rotation of the surfaces projected into the $\left(x_{w},x_{n}\right)$ evaluation plane, *θ*; and relative gradients in *z* along a set of rays, $\delta z_{r}.$ *ρ* estimates the displacement of the simulated surface in $\left(x_{w},x_{n},z\right)$ space relative to the experimental, due to either or both components of the model. *θ* accounts for different amounts of rotation over the surfaces, due to tilting of the planar component of the producing function. $\delta z_{r}$ captures differences in the “bending” of the surfaces, due to either or both components of the model. We discretized the shapes using 10 mesh points placed at the same relative distances from the peak along each of six ray segments of fixed slopes (the “relative mesh points”). The segments are bounded by the peak and the edges of the evaluation plane. Non-Mo17 surfaces had ray segments at slopes {0,0.05,0.099,0.175,0.32,0.75} relative to the origin. For Mo17, the slopes were {0,−0.05,−0.099,−0.175,−0.32,−0.75}. This discretization divides the surface into nine adjacent bands whose tilts and twists reflect shape changes in that region.

R code to generate mesh points, compute comparisons, and plot heatmaps is in File S14. We used the R packages superheat and viridis for the heatmaps (Barter 2017–present).

### Data and Code Availability

All supplemental files (input data, SAS analysis code, outputs, supplemental methods, supplemental results, and outputs are available from FigShare at https://figshare.com/s/3ef69b44d24d0953d625. Code for modeling, fits, and simulations is on GitHub at https://github.com/tonikazic/univariate_dose_response.git in a public repository.

## Results

### Effect of Stress Combinations on Parental Inbreds

**Figure 1:**
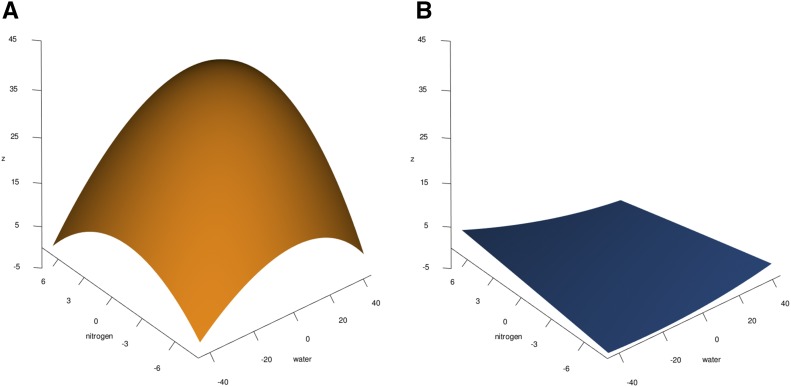
*Parental Inbred Response Surfaces to Combined Drought and Nitrogen Deprivation*. A quadratic surface was fit to the measured trait of differences in plant height (*z*) and is shown for each parental inbred on the same scales. (A) The B73 inbred response surface; (B) the Mo17 response surface.

Table 1 shows the parameter values obtained by fitting Equation 1 to the experimental data for the parental lines (first and last rows). The response surfaces generated using these parameters are plotted in Figure 1 for the parental lines. The parental inbreds exhibited different responses to combinations of water and nitrogen deprivation, with B73 showing more variation in its response surface than Mo17. The plots are rotated to show the most severe stresses in the front center corner, placing the normal full-water and full-nitrogen combination in the back. The intersections of the surface with the nitrogen-height ($\left(x_{n},z\right)$) and water-height ($\left(x_{w},z\right)$) planes define the phenotypic response at constant amounts of water and nitrogen, respectively, corresponding to the discrete partial differentials $\delta z/\delta x_{n}\;\text{and}\;\delta z/\delta x_{w}.$ B73 exhibited a domed surface, with the peak at moderate amounts of water and nitrogen (Figure 1A). This surface is convex upward in the sense that it opens downward toward negative values of *z*; and has a relatively high, and highly curved, peak (large $z_{\max}$ and small discrete curvature), that lies in the region of relatively high nitrogen and water (Sullivan 2006). While B73 declined under the most severe conditions (front center corner), it showed modest growth under both moderate drought and very low nitrogen (*e.g.*, the maximum of $\delta z/\delta x_{w}$ at the surface’s right edge) and high drought and middle nitrogen (*e.g.*, the maximum of $\delta z/\delta x_{n}$ at the surface’s left edge). The worst condition was minimum water and maximum nitrogen (rear left corner).

In contrast, Mo17 had little growth change under any stress (Figure 1B). Its surface is a very shallow trough, or is concave upward; the nadir, $z_{\min},$ is small; its discrete curvature is larger; and the peak lies on a corner. For Mo17, reducing nitrogen affected growth slightly more severely than reducing water (compare the slopes along the left front and right front faces of Figure 1B. We investigated whether this small difference in Mo17’s change of heights could be due to its decreased overall growth. We compared the initial plant heights of B73 and Mo17 and found no significant differences between them (P=0.18). When we compared the inbred plant heights after deprivation, we found that the B73 plants had more growth and greater differences between treatments than Mo17 (comparing the factors inbred, nitrogen level, water level, and inbred by nitrogen level, at P<0.05; the numerous sample sizes and confidence intervals are reported in File S9). But when we scaled the differences in height to adjust for the smaller Mo17 plants at the beginning of the experiment, plant growth during the experiment was more pronounced for Mo17 in mid-level nitrogen and low water-level treatment combinations. In contrast, B73 growth was typically greater when more water was available. This indicates that the Mo17 inbred line is less sensitive to drought provided at least some nitrogen was present. Comparisons at very low nitrogen levels did not exhibit any trend toward differences between parental inbreds (Figure S8). For both B73 and Mo17, the slopes of the four lines intersecting each pair of the surfaces’ corners are different, indicating the plants’ responses vary with extremal stress combinations. Mixture toxicity models with two shape parameters, < a and b> for water and nitrogen, were used to analyze the shape differences in the parental B73 inbred. (These two parameters are *not* the same as the *a* and *b* of Equation 2, and we have typeset them in a different font than is normally used in mixture toxicity papers to emphasize this distinction.) B73 had the best fit to a dose-ratio surface. < *a* and *b* > indicating that the antagonistic effect of combined stresses is caused mainly by nitrogen deprivation. This is consistent with the curvature of the discrete partial differentials at the edges of the B73 response surface in Figure 1A: $\delta z/\delta x_{w}$ is more sharply curved and has a higher local maximum than $\delta z/\delta x_{n}.$

### QTL That Change Phenotypic Response Surfaces

**Figure 2:**
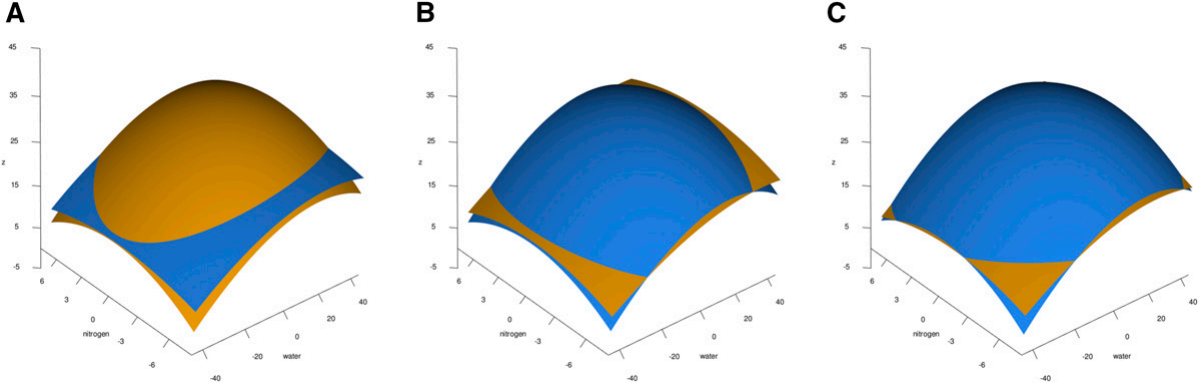
*Loci with Significant Effects on the Phenotypic Response Surface*. Three QTL with a Sidak-adjusted significant interaction surface for differences in height were identified. At each QTL, the trait data were fit with a quadratic response surface separately for each allele. The B73 allele is shown in orange and the Mo17 allele in blue. Each panel shows the QTL’s location (chromosome bin and marker range indicated above) and surface fits for the allele differences at the locus. (A) Surfaces for QTL1, *gpm906a – IDP2465*; (B) surfaces for QTL2, *umc1838a – mmp22*; and (C) surfaces for QTL3, *ufg71 – IDP1681*.

Chromosomal loci with significant interaction *P* values for the response surface were fit to nitrogen deprivation and drought. The interaction effect is visualized as the intersections between the surfaces in the illustrative plots in Figure 2. The interaction between marker allele and each stress combination covariate was fit with a standard linear model, and the parameters derived from the model were those used in mapping the QTL. We found three highly significant QTL that changed the response surface fitted from the data on all lines and an additional 50 QTL when false discovery rate-adjusted *P*-values less than 0.05 were considered. The three QTL with *P* values below the experiment-wise Sidak-adjusted significance threshold of 0.05 are shown in Figure 2. Table 1 shows the parameter values obtained by fitting Equation 1 to the data for the smoothed QTL response surfaces.

For all three QTL, the response surface for the Mo17 allele is upwardly convex, a shape resembling that of the B73 allele and parent, and very different from the upwardly concave shape of the Mo17 parental surface. The three QTL’s alleles differ from each other and from parental inbreds in many details, including the magnitudes of *z* over their surfaces, the relative magnitudes of the B73 and Mo17 surfaces, and the value and position of $z_{\max}.$ The Mo17 allele’s surface for QTL1, shown in Figure 2A, is pushed upward along the *z* axis, far above the range of the Mo17 parent’s response in Figure 1B. For extremal combinations of water and nitrogen, changes in the growth of the Mo17 allele exceed those for the B73 allele. QTL2’s and QTL3’s Mo17 surfaces lie mostly above those of their B73 alleles. For these loci, the B73 alleles exhibit better performance under extremal conditions (see Figures 2B and 2C). Thus, the phenotypic response surface can differ in shape and magnitude within the population, and surface shape can be quite different than the parental response surface in offspring carrying some QTL allele combinations. Because the experimental design was optimized to detect marker-stress treatment interactions using Equation 1, these QTL display a crossover interaction between the allele surfaces. Like their parents, none of these alleles have surface corners that lie on parallel lines.

The differences in surface shape between allele fits in all three QTL, with highly-domed surfaces that have increased combined stress peaks in the middle of the response surface, indicate that nitrogen and water stress have nonlinear effects on plant growth. These B73-like surface shapes indicate that the better-performing allele at mid-range combined stress is typically not the allele that provides best performance under extreme conditions. The highest water and nitrogen input conditions, which might naïvely be assumed to support the most growth, exhibit less growth and could be favored by a different allele than the mid-range combinations.

### The Shapes of the Alleles’ Response Surfaces

The most distinctive and robust feature of the phenotypes is the shapes of the surfaces, rather than their absolute placement in $\left(x_{w},x_{n},z\right)$ space. The shapes show the patterns of the alleles’ responses to the stresses, and are less sensitive to the effects of errors due to small sample sizes. The smoothed experimental surfaces fall into four *nondisjoint* categories:

**domed** more sharply domed, highest amplitude surfaces with peaks in the high nitrogen, high water region (B73 and QTL1-B73);
**hybrid** higher amplitude domed surfaces with the peaks displaced from the high nitrogen, high water corner toward the center of the water-nitrogen plane (QTL2-Mo17 and QTL3-Mo17);
**shoulder** lower amplitude surfaces, with lower peaks on the high water edge that slope more gradually downward as nitrogen decreases (QTL1-Mo17, QTL2-B73, and QTL3-B73); and
**trough** a very low amplitude trough with a peak at the lowest water and highest nitrogen corner (Mo17).

These categories are illustrated by examining the positions of the peaks in $\left(x_{w},x_{n},z\right)$ (Table 2), and by projecting the surfaces into the water-nitrogen plane (first and third columns of Figure 5).

**Table 2:** *Comparison of Numerical Values of the Experimental Peaks’ Positions and the Nondisjoint Shape Classification*. These values were obtained from the surfaces generated using Equation 1 and the fitted parameter values of Table 1.

	*domed*		*hybrid*		*shoulder*			*trough*
*criterion*	B73	QTL1-B73	QTL2-Mo17	QTL3-Mo17	QTL1-Mo17	QTL2-B73	QTL3-B73	Mo17
$x_{w}$	28.5	31.5	24.0	25.0	37.0	42.0	42.0	−42.0
$x_{n}$	4.5	3.5	3.0	3.0	5.0	5.0	6.0	7.5
$z_{\text{max}}$	37.6	34.5	34.2	35.2	30.9	33.3	32.4	4.5

We emphasize that the membership of the middle categories depends on the classification criteria. If one considers just the position of the peak in the $\left(x_{w},x_{n}\right)$ plane, then B73 and QTL1-B73 would form the domed class, and QTL2-Mo17 and QTL3-Mo17 would form the hybrid class. Weighting the peak’s position in *z* more than in $\left(x_{w},x_{n}\right)$ would classify only B73 as domed and shift QTL1-B73 into the hybrid class with QTL2-Mo17 and QTL3-Mo17. Binning the $z_{\max}$ more coarsely would eliminate the hybrid category altogether. Adding other criteria, singly or in combination, might further change the classification.

### Annotations of Gene Function in QTL Regions

**Figure 3:**
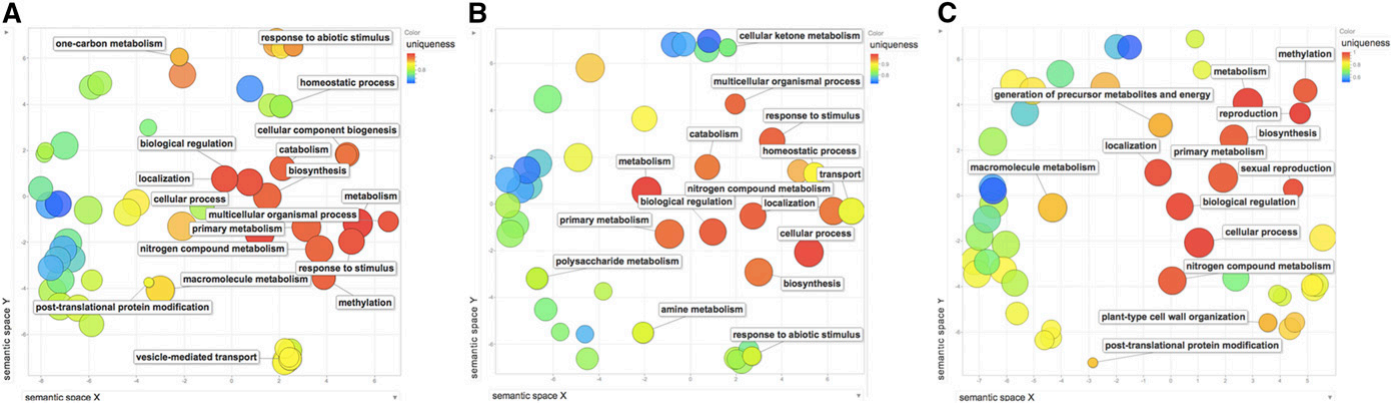
*Gene Ontology Annotations for QTL*. All known genes in each QTL region were scanned for significant annotations. GO process annotations are shown. Annotations were ordered by semantic similarity (Supek *et al.* 2011), with single genes under the QTL having higher uniqueness (redder shades). (A) QTL1, bin 1.06, bounded by markers *gpm906a* to *IDP2465*; (B) QTL2, bin 1.08, bounded by *umc1838a* to *mmp22*; and (C) QTL3, bin 9.03, bounded by *ufg71* to *IDP1681*.

Gene annotations under QTL provide a qualitative new data type that can provide additional context to the mapping of chromosomal loci. Annotations such as ”response to abiotic stress” in the two QTL on Chromosome 1 (Figure 3A and 3B) are consistent with our identification of these QTL as important for response to drought and nitrogen fertilizer. QTL3 in bin 9.03 does not have unique annotations in stress response (Figure 3C); this may indicate that a novel gene type is responsible for the causal allele difference at this locus. The marker with the smallest *P* value within the QTL1 region was *IDP168*, which tags gene *GRMZM5G828396*. This gene is annotated as a basic Helix-Loop-Helix (BHLH) transcription factor. The marker with the lowest *P* value in the second QTL interval was *umc1446*, which tags gene *GRMZM2G162508*; this gene is annotated as a polyketide-synthase-like protein. The marker with the smallest *P* value in the third QTL in bin 9.03 was *mmp17b*, which is between the genes *GRMZM2G538859* and *GRMZM2G093187*; neither gene model has assigned annotations.

### Modeling the Response Surfaces With a Producing Function

What is the simplest physiological model that produces the phenotypes? If one assumes the phenotypes are produced by a single network in the plant, then the corresponding producing function is the most parsimonious network. Tuning the function’s parameters to reproduce the observed phenotypic points is equivalent to tuning the network’s parameters, rather than its connectivity. The phenotypic points form an *n*-dimensional space, with each phenotype a point in this space. The greater the range of observed phenotypes, the more the phenotypic space is sampled, and the more constraints an hypothesized function must satisfy. Such a function can also predict novel phenotypic points that lie in the space.

Equation 1 is not the best choice for the producing function. Its large constant, lumped together as ℓ in Table 1, shifts the surfaces along the *z* axis without modifying their shapes. It assumes the hypothesis — an interaction between water and nitrogen — via the hyperbolic paraboloid term. Finally, it has a fairly large number of terms.

We therefore searched for a simpler function that would reproduce the surfaces’ shapes without explicitly assuming an interaction between the inputs. The single, sharp global maxima of the B73-like surfaces immediately suggested an elliptical paraboloid. This is the simplest function that generates a single peak; changing the sign of one parameter flips the peak to produce Mo17’s trough. An orthogonal projection of the paraboloid into the $\left(x_{w},x_{n}\right)$ evaluation plane gives an ellipse, whose ratio of major and minor axes reflect the relative weighting of the water and nitrogen inputs for that phenotypic point. We considered other functions that generate peaks, but they are structurally more complicated, harder to flip, and need more assumptions that are more difficult to justify. For example, a two-dimensional Gaussian function is structurally more elaborate, and flipping requires the reciprocal of the Gaussian. Periodic functions, such as transcendental or Bessel functions, would have forced us to assume either that their other peaks lie outside the evaluation interval or that the functions are severely damped.

However, three asymmetries in the phenotypes indicate the producing function is not just an elliptical paraboloid. First, the maxima are not where they would be for an elliptical paraboloid, at (0,0,z). Second, the surfaces are tilted: intersecting each surface with the four planes perpendicular to the evaluation plane yields a set of different surfaces. Third, the surfaces are not symmetric around the peaks, but distorted. While the peak can be shifted by adding a constant along $x_{w},$ $x_{n},$ or both, tilting and distorting the surfaces requires the addition of another component to the producing function. We experimented with many possibilities for this second component, including exponential and transcendental functions and operators to combine the first and second components, and found the simplest approach was to add a plane.

Thus, the simplest producing function is that shown in Equation 2, reproduced here:

$$z=\underset{\text{elliptical}\;\text{paraboloid}}{\underset{︸}{c\left(ax_{w}^{2}+bx_{n}^{2}\right)}}+\;\underset{\text{plane}}{\underset{︸}{dx_{w}+ex_{n}}},$$

where *z* is the difference in height; $x_{w}$ is water; and $x_{n}$ is nitrogen. a and b modify the paraboloid’s size, shape, and weighting of input water (*a*) and nitrogen (*b*). *c* modifies the paraboloid’s size and shape and flips it: c<0 makes the surface convex upward like the B73-like surfaces, while c>0 produces the concave upward surface of Mo17. d and e tilt the plane along the water and nitrogen axes, respectively. This shifts, stretches, rotates, and tilts the paraboloid to change its shape with a set of affine transformations. The relative weighting of the paraboloid and plane components is controlled by all five parameters: c(a+b)>d+e emphasizes the paraboloid features of the surface. The relative weightings of water and nitrogen within each component are different (a and b, d and e), and independent of the weightings of the two components.

**Figure 4:**
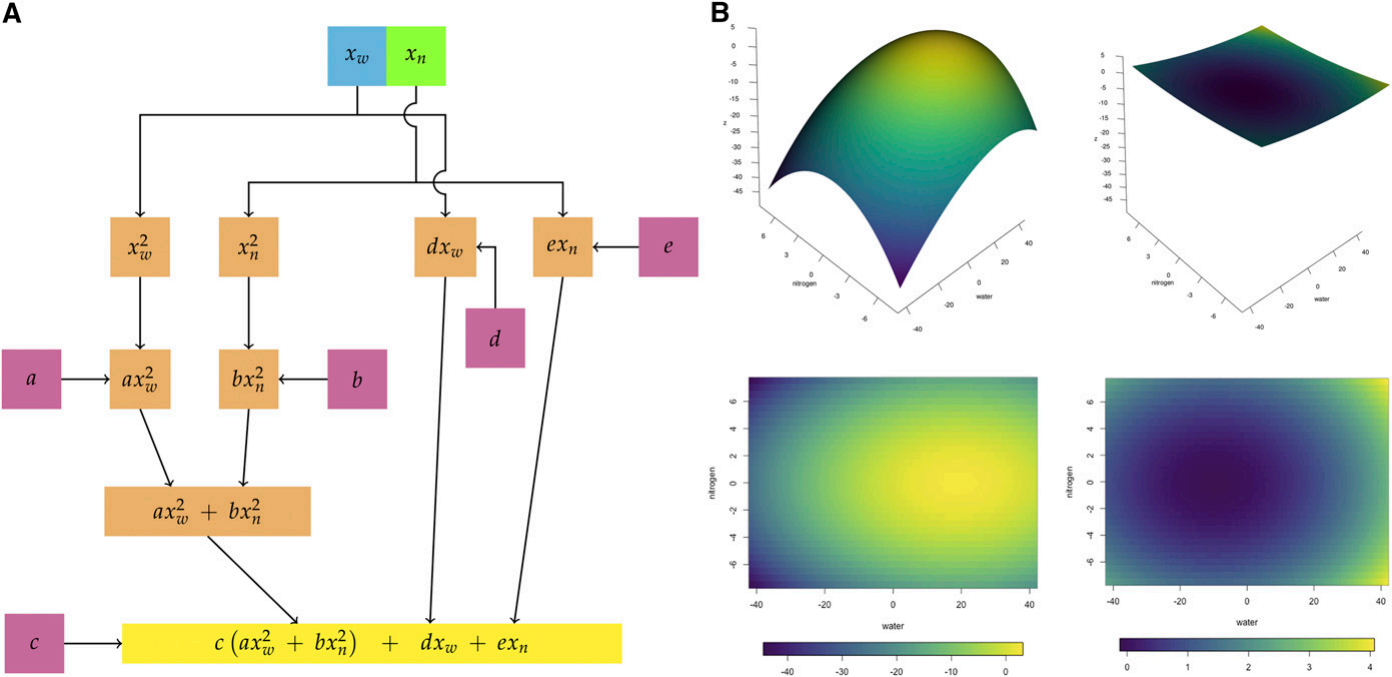
*Modeling of Response Surfaces with the Producing Function (Equation 2)*. (A) The producing function drawn as a network. The elliptical paraboloid component is on the left, and the planar component on the right. Each term in the function is a node; the operators are edges. The parameters of the producing function (Equation 2) are in magenta; results of mathematical operations on the input nodes are shown in orange. The final equation is in yellow. (B) Sample simulated response surfaces and their projections into the $\left(x_{w},x_{n}\right)$ plane. (left) B73-like response surface, (a,b,c,d,e)=(0.260,0.305,−0.175,2.575,0.500); (right) Mo17-like response surface, (a,b,c,d,e)=(0.0330,0.4250,0.0030,−0.0025,0.5500). The color scale for each is the same for its surface plot and projection.

We tested the model by deleting terms and by varying the values of the parameters. Both components are essential to reproducing the experimentally observed surfaces. Omitting the elliptical paraboloid makes it impossible to reproduce any experimental surface, since all have domes or troughs; and omitting the plane makes all of the surfaces symmetric about the major and minor axes of the paraboloid, placing the maxima and minima at (0,0,z). Each term in the equation corresponds to a node in the network, drawn in Figure 4A. The simulated surfaces in Figure 4B show good qualitative agreement with the experimentally observed surfaces of the B73 and Mo17 parental inbreds in Figures 1A and 1B. Thus, the phenotypes we observe are products of the entire network.

### Parameter Estimation for the Producing Function

How well does Equation 2 reproduce the shapes of the alleles’ response surfaces? To answer this question one must first estimate the model’s parameters for each allele. However, the surfaces’ nonlinearity entails several trade-offs that affect the accuracy of the estimates. The edges of the surfaces were under-sampled to optimize the experiment for peak detection. By omitting a hyperbolic paraboloid, the model cannot reproduce the apparent nonplanar, twisted quadrilateral of the surfaces’ corners. Circumventing those issues by fitting with data near the peaks makes the estimates more sensitive to the smoothing errors inherent in flatter peaks, and inadvertant affine transformations of the surfaces due to slight changes in a,b,d,or e. Linear regression is simpler, faster, and more likely to converge than nonlinear methods, but will exaggerate the peak regions since their *z* values are so much greater.

With these caveats, we estimated the model’s parameters for each line by linear regression to Equation 3. We used a set of absolute mesh points that sampled the central, best-determined parts of the smoothed experimental surfaces. We preset *c* to {−1,1} to simplify estimation, and estimated parameters using and omitting the peaks. Table 3 summarizes the values of a,b,d,and e, and the square root of the solution norm scalar errors, *s*, for each experimental surface. The residual norms for all the parameters were 0. In general, the estimated values distribute the numerical weight more evenly among the four parameters than the fits to the regression model of Equation 1, shown in Table 1. The exception is QTL3-Mo17, where nearly all of the numerical weight is concentrated in *e*. The parameters shown were estimated by including the experimental peaks. Judged by *s*, omitting the peaks slightly degraded the quality of the estimates for all lines except Mo17 (Figure S10).

**Table 3:** *Parameter Values*, *Errors*, *Sign Patterns*, *and Shape Types for Experimental Surfaces Fit to Equation 3*. The absolute experimental mesh points used in these estimates included the peak of each surface. The value of *c* was preset to produce the appropriate convexity. *s* is the square root of the solution norm. The residual norms were all 0.

*line*	*a*	*b*	*c*	*d*	*e*	*s*	sgn(a)	sgn(b)	sgn(d)	sgn(e)	*shape*
B73	−0.0463	−1.8005	−1	−0.0765	1.3540	146.0321	−	−	−	+	domed
QTL1-B73	−0.0429	−0.5926	−1	−0.3134	−0.9222	127.2874	−	−	−	−	domed, ambiguous
QTL2-Mo17	−0.0332	−0.4362	−1	−0.3681	−2.9073	134.1696	−	−	−	−	hybrid
QTL3-Mo17	−0.0636	0.0039	−1	−0.5199	−4.0788	112.6761	−	+	−	−	hybrid
QTL1-Mo17	0.0164	−0.6201	−1	1.2777	1.8758	89.4613	+	−	+	+	shoulder
QTL2-B73	0.0268	−0.4119	−1	1.7254	1.7119	81.4159	+	−	+	+	shoulder
QTL3-B73	0.0278	−0.4450	−1	1.6600	1.0073	83.2248	+	−	+	+	shoulder
Mo17	0.0259	0.2164	1	1.7406	−0.7203	34.1571	+	+	+	−	trough

**Figure 5:**
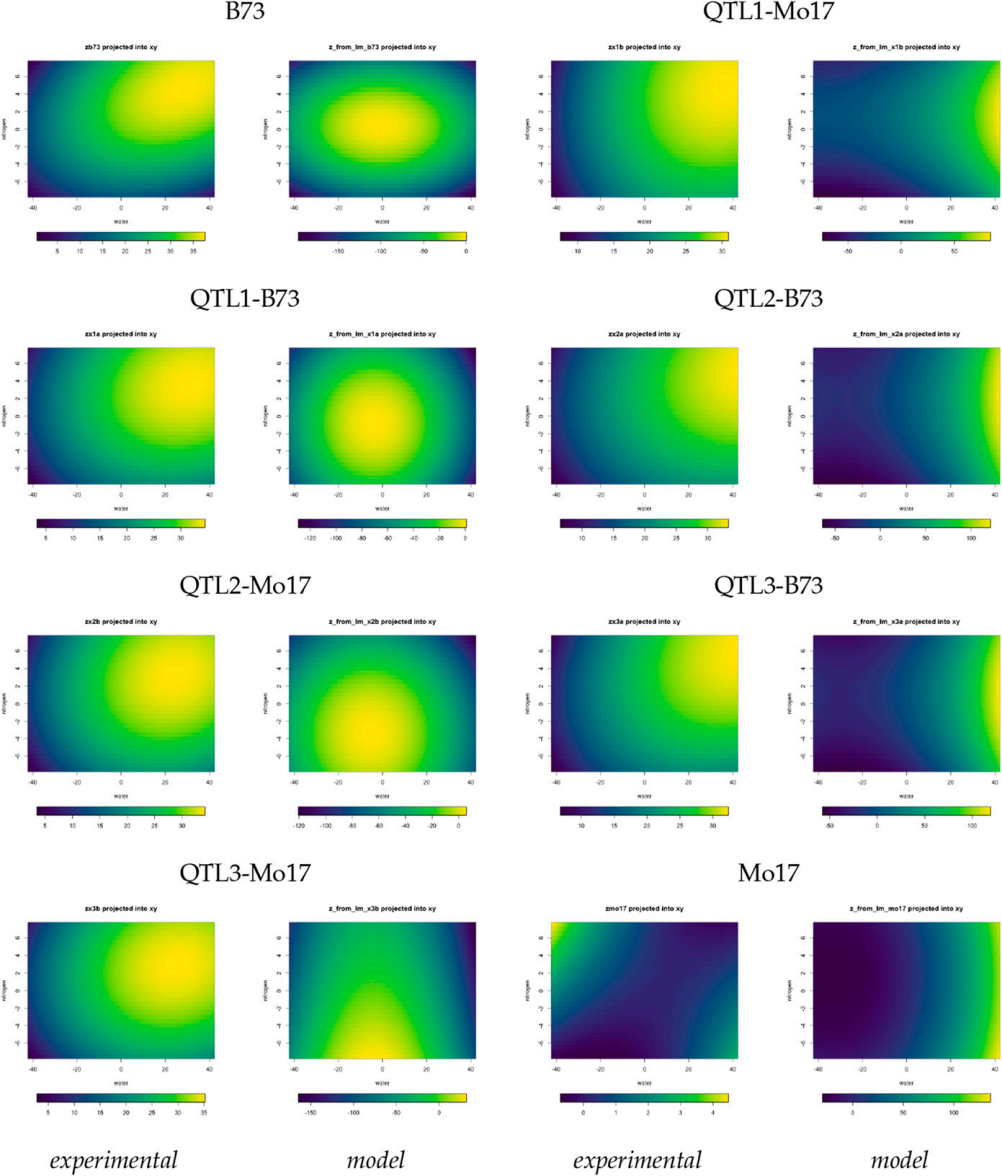
*Generated Experimental and Model Surfaces Projected into the Evaluation Plane*. Experimental surfaces were generated using Equation 1 and Table 1, marked “experimental”. Model surfaces were generated using Equation 2 and Table 3, marked “model”. The left two columns show the domed and hybrid shape categories, and the right two columns the shoulder and trough categories. Each surface’s scale spans its maximum and minimum: bluer shades are near the minima and yellow the maxima.

The right side of Table 3 summarizes the patterns of parameter signs, and compares these to the independently derived classification of the surfaces’ shapes shown in Table 2. Grouping by signs makes it more visually obvious how the surfaces in Figure 5 connect to the parameters of the producing function, and emphasizes the overlap among the categories. The sign patterns match the shape classification shown in Table 2 except for QTL1-B73 and QTL3-Mo17. QTL1-B73’s classification strongly depends on how the classification criteria are weighted, and its estimated values for b and d are much closer to those for the hybrid shape of QTL2-Mo17 than B73’s. QTL3-Mo17 is a hybrid surface based on the peak position in $\left(x_{w},x_{n},z\right)$ (Table 2), the projection into the evaluation plane (Figure 5), and its estimate for *e*, but has a distinct sign pattern that reflects the small estimated value of *b*. Both the sign pattern and the estimates of a,b,d, and e for the shouldered lines are much more internally consistent.

### Comparison of Experimental and Simulated Surfaces

To see how well the model reproduces the shapes of the phenotypes, we compared the smoothed experimental response surfaces to those generated by the model. The experimental surfaces were generated by Equation 1 and Table 1, while the model’s surfaces used Equation 2 and the parameter values from Table 3. Figure 5 shows these surfaces projected into the $\left(x_{w},x_{n}\right)$ evaluation plane. They are plotted to show each surface’s amplitude by using its minimum and maximum to set the scales. The producing function reproduces the major shape features of the phenotypes. All four shape categories are generated with the correct membership and ambiguities. The surfaces illustrate the caveats of using linear regression for nonlinear phenomena for the values in Tables 1 and 3. Relative to the generated experimental surfaces, all the model surfaces are rotated counter-clockwise and their amplitudes exaggerated. Model surfaces are translated relative to the evaluation plane:domed and hybrid surfaces toward the low-nitrogen edge; the shouldered surfaces toward the high water edge; and Mo17 toward the low nitrogen, high water corner.

We assessed the intrinsic shapes of the surfaces using three types of similarities, computed on a mesh of points that extend from the peaks leftwards and downward. Unlike the absolute mesh points used in the linear regression that are very sensitive to affine transformations of the surfaces, these relative mesh points are placed at constant relative distances along a set of ray segments that cover the lower left quadrant of the surfaces, and are adequately robust to translation of the surfaces relative to the evaluation plane. Three similarity scores were computed. *ρ*, the signed Euclidean distance, evaluates the displacement of the model surface in $\left(x_{w},x_{n},z\right)$ space relative to the experimental due to either or both components of Equation 2. *θ*, the rotation angle between the surfaces projected into the $\left(x_{w},x_{n}\right)$ evaluation plane, accounts for different amounts of relative rotation due to tilting the planar component. $\delta z_{r},$ the gradients of relative discrete differences along the rays as one moves away from the peaks, captures differences in the “bending” of the surfaces due to either or both components.

**Figure 6:**
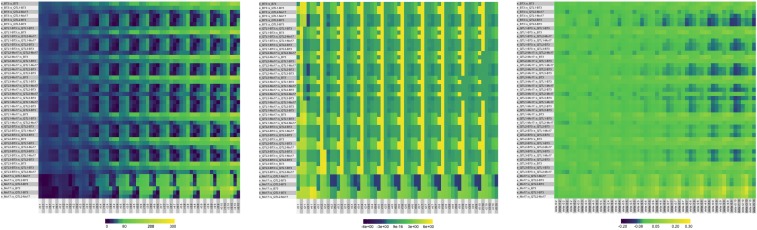
*Heatmaps of Surface Similarity Scores* ρ, θ, *and $\delta z_{r}.$* The pairwise comparison of all 60 relative mesh points for each pair of experimental (e) and simulated surfaces (s) are the rows; the values for the pair of mesh points are the columns. The columns are ordered by concentric rings around the experimental peaks, so that the leftmost six columns are closest to the peak (:1) and the rightmost six furthest away (:10). Within each set of six columns, the ray segments (r0,r1,…,r5) are arranged in order of increasing slope (non-Mo17) or decreasing slope (Mo17). For $\delta z_{r},$ bluer colors correspond to steeper gradients for the simulated surfaces, relative to the experimental ones, and yellower colors the opposite. The scales are heatmap-specific.

We compared the shapes’ similarities for each pair of generated experimental (e) and model (s) surfaces at each corresponding pair of relative mesh points. The QTL3-Mo17 and Mo17 parameters produced surfaces that were displaced too far in the evaluation plane to be included in the comparison. Values for $\rho ,\;\theta ,\text{and}\;\delta z_{r}$ for each pair of comparisons are shown as heatmaps in Figure 6. In the heatmaps, each experimentally-derived surface (e) is compared to all available model surfaces (s), beginning with its model sibling. Each comparison is a row, and the columns are ordered to form concentric rings around the peaks. The first ray segment in each ring is parallel to $x_{w},$ the water axis, and the last nearly parallel to $x_{n},$ the nitrogen axis. Bluer shades indicate higher similarity for *ρ*; for *θ* and $\delta z_{r},$ the greener shades indicate higher similarities. For $\delta z_{r},$ bluer shades indicate the simulation’s gradients are steeper compared to the experimental; yellower shades signal the simulation’s gradients are shallower.

Shape reproduction by the model is good, with different pairs of surfaces showing reasonable accuracy over their entirety for the different similarity scores. Blocks of similar blue and green colors dominate all three scoring matrices, and correspond to the nondisjoint shape categories. The same experimental surface was often fit equally well over most of its surface by multiple simulations, as judged by two or more criteria. For example, generated experimental shouldered surfaces QTL1-Mo17, QTL2-B73, and QTL3-B73 were best emulated by non-Mo17 surfaces; B73 was poorly emulated; and Mo17 was never well emulated by simulations of the non-trough surfaces. Occasionally the self comparisons were not the best fit, suggesting we did not overfit the model’s parameters. For example, the shouldered surfaces were less well emulated by their simulated selves, compared to B73. Changes in gradients along the shapes were usually least for the experimental surfaces compared to their simulated siblings. All simulated non-Mo17 surfaces tended to descend more sharply toward the tails of the surfaces in several comparisons. Mo17 descended more shallowly when compared to any other simulations, as one would expect.

Some nuances deserve highlighting. As expected, the accuracy of shape reproduction is not uniform over the surfaces for any similarity score, and the three scores often differ for any given comparison. Reproduction is better nearer the peaks and for the more central ray segments. The regression to Equation 3 was based on those regions, but the performance of the model outside them is often almost as good for many comparisons. The scores are still somewhat sensitive to affine transformations. For example, the repeating yellow stripes in *θ* for ray segment r1 in Figure 6 are an artifact of the way *θ* is calculated. As the peaks from which they are drawn shift in the $\left(x_{w},x_{n}\right)$ plane, the absolute lengths of the ray segments compared increases, increasing the apparent rotation. Thus, the maximum rotation we observe along r1 reflects greater length differences in the r1 ray segments relative to the others.

## Discussion

We observed that the parental B73 and Mo17 inbreds show different responses to combined stresses (Figure 1). The position of maximum response and the surfaces’ shapes differ markedly among the parental and recombinant lines (Figure 2 and Table 2). All three of the QTL we identified have better performance for one allele in the mid-range combined stress, at moderate levels of water and nitrogen. Mo17 is the least responsive of all the lines, with a flat trough responding to all water-nitrogen combinations. In constrast, lines with Mo17 QTL in mixed RIL backgrounds have responses that show much greater, more B73-like amplitudes and convexities. All Mo17 QTL exhibit B73-like convexity, but their other effects on the baseline B73 response vary by QTL: QTL2-Mo17 and QTL3-Mo17 lift the response above their B73 alleles. The responses of the B73 alleles are damped, and shifted by the addition of Mo17 germplasm in the background across all environments (Table 2). These differences in the response surfaces illustrate the effects of multiple segregating genes for this population in combinations of water and nitrogen. The QTL we identified have contrasting and intersecting response surfaces. To estimate an effect size for these QTL, we would need a way to express the shape differences in multiple dimensions for fitting. Simply calculating the slope along a slice of the surface, or at $z_{max},$ would not capture the full effect of the QTL and would mis-estimate the effect size.

One interpretation of the nonlinear response surfaces is that they arise from epistatic interactions between the QTL and other modifier loci in the background. Antagonistic, damped responses to combinations of stresses were previously observed in this IBM population in an experiment measuring changes in height under combinations of UV and drought, which identified different QTL than we see here for drought and low nitrogen (Makumburage *et al.* 2013). Our results are consistent with the importance of nitrogen for growth for modern corn lines (Hallauer *et al.* 2010) and the ability of lower levels of nitrogen fertilization to ameliorate the effect of severe drought under certain conditions (Sadras and Richards 2014). For example, the B73 MixTox analysis indicates that low nitrogen “over-shadows” drought; in low nitrogen, having additional water available does not improve growth. Since combinations of other stresses may have different genetic control, dose response analyses for more stress combinations, and perhaps more complex surface fits such as dose-ratio (Jonker *et al.* 2005), would be needed.

While epistatic interactions are common, they are difficult to detect in small mapping populations using combinatorial or variance component methods (Mäki-Tanila and Hill 2014; Huang and Mackay 2016). Although we weighted our statistical model (Equation 1) for overall polygenic similarity, we did not have sufficient data to test for specific epistatic interactions. However, it is likelier that any epistasis one might deduce from a linear model is instead the result of attempting to fit a linear model to a nonlinear response such as those seen here, or mis-assigning the meaning of the various variance components (Sailer and Harms 2017; Huang and Mackay 2016). Instead, one would solve for the nonlinear function and its matrix of coefficients. The latter captures both the interactions among genes and their entailed products and reactions; and the magnitudes of those interactions (the effect size). Such a function and its matrix define a physiological model of the network that, as an entirety, produces the phenotypic collection. Paths through the network select subsets of terms from the full nonlinear model, fragmenting the phenotypic collection into multiple approximations that can appear as epistasis. Epistatic interaction terms can also arise from linear transformations of nonlinear responses, which decouple the network into a matrix of connecting interactions and a matrix of the interactions’ magnitudes. Better nonlinear models would fold these apparent epistases into the inherent nonlinearity of the response.

Nonlinear changes in growth in response to combined stresses form a complex phenotype. Just from the surfaces fit to the experimental data, one can rule out an unbranched network sensitive to both water and nitrogen, because the response’s peak does not scale with the sum or product of the inputs. Similarly, one can exclude two completely independent nodes, one for each input. Instead, the two inputs interact so that the response varies as a function of both: the phenotypic collection is produced by the action of the entire network, rather than just paths through it (Figure 4A). The nonlinear producing function of Equation 2 defines this interaction as the sum of two components, each of which is sensitive to both water and nitrogen. It successfully accounts for all the important qualitative features of the experimentally observed phenotypic collection without assuming the large constants that dominate the solution in the QTL analysis (Equation 1 and Table 1). Surfaces generated using parameter values obtained by linear regression approximated the shapes of the experimental surfaces well, but were shifted downward in three-dimensional space (Figures 5 and 6). The heatmaps illustrate how much the surfaces can change with the parameter values. Slightly tilting the plane component of Equation 2 can strongly shift and rotate the position of the peak in $\left(x_{w},x_{n},z\right).$ This suggests the model is quite sensitive to variation in *d* and *e*, and is consistent with our simulations (data not shown).

Unlike crop models that frame the organism’s physiology as a system of equations that involve large numbers of parameters that may not be directly measured (Cooper *et al.* 2016; Technow *et al.* 2015), the producing function is a simpler, more coarsely-grained model that approximates the behavior of the system. It explicitly treats the parameters as fundamental model components that adjust the organism’s physiological response to input water and nitrogen. Environmental perturbations, such as varying the available water and nitrogen, change the intervals over which the producing function is evaluated by the plant. Genetic perturbations, such as the alleles of the QTL identified in this work, delimit different regions in the phenotypic space in which possible responses lie. Of course, considering additional phenotypes or phenotypic dimensions might necessitate changing the function.

The usual objective of QTL experiments is to isolate loci that exert a change on a single dimension of a phenotype, such as the mean (Lynch and Walsh 1998). The assumptions are that the mappings between QTL and the parameter space, and between the parameter space and the phenotypic space, are one-to-one and onto; that the association function is linear and additive; and that one QTL can be freely exchanged for another with an effect of similar magnitude. However, our data clearly show none of these assumptions hold. The phenotypic collection we observe falls into four overlapping categories, and the close similarities of the phenotypic points within each category, and the overlap between the domed and hybrid categories depending on the classification criterion, suggest that the parameter values governing them can fall into rather broad ranges. This is a hallmark of sloppiness in model systems that breeders commonly call equifinality, and statisticians call “parameter nonidentifiability” (Transtrum *et al.* 2015; Luo *et al.* 2009; Hartung 2014; Hines *et al.* 2014). This interpretation is supported by extensive simulation experiments: so far, we have been unable to identify unique combinations of parameter values that determine each phenotypic point. The parameter ranges for the observed phenotypic points are not disjoint, another characteristic of sloppy systems (data not shown) (Transtrum *et al.* 2015). Thus, our data divide both the parameter and the phenotypic spaces into nondisjoint subspaces, and confirm the mapping is many-to-many.

Our current approach detects single QTL that control shifts among points in the phenotypic space, changing entire response surfaces. How could one detect portions of the network that affect different aspects of the phenotypic collection? For example, are there QTL that influence just the elliptical paraboloid or that restrict which shape categories can be reached from another? One would need to look for sets of QTL that jointly influence phenotypic features: these phenotypic subspaces, identified by specific loci, correspond to a higher resolution view of portions of the system. The many-to-many mappings among markers, the parameter space, and the phenotypic space would also have to be explicitly considered. We can think of two ways to approach this problem. The first way is to ask for markers that jointly affect subsets of parameters, shifting the phenotypic points from one phenotypic subspace to another. The overlapping categories of shapes we observe may reflect genuine nondisjointness in phenotypic space or missing dimensions that would separate the categories in a higher dimensional space. The second approach is to ask how many distinct groups of phenotypic points are in the collection by unsupervised clustering of all the experimental units. The membership of the resulting clusters would not necessarily be coincident with the genotypic or stress labels, nor would they necessarily be disjoint. One would then ask for intersecting sets of loci within and across all clusters, which allows many-to-many mappings. The clusters in phenotypic space correspond to allowable states of the underlying system and its producing function, joined by appropriate transitions among the clusters. The sets of loci may not be mutually disjoint, and subspaces of the parameters will differ in their sloppiness. These approaches could be tested first by simulations that randomly assign markers to different simulated surfaces.

The success of this endeavor hinges first on the ability to recognize similarities among high-dimensional surfaces. The technique we used of scoring similarities by multiple criteria over a set of mesh points can be readily extended to higher dimensional surfaces, and used for clustering phenotypic points, optimizing parameter searches, and genetic mapping. For example, looking across all three scoring matrices in Figure 6 would produce a vector in 179-dimensional space. The second condition is the ability to fit parameters to nonlinear phenomena. Estimating parameters by fitting is more challenging with nonlinear phenomena. Two questions arise: what should be fitted? and how should it be fit? The first question is the fit criterion. We tested a wide variety of objective functions, singly and in combination, to describe the surfaces through a set of proxies that are faster to compute than the scores over the mesh points. None effectively discriminated among somewhat similar surfaces. This is not surprising in retrospect, given the many-to-many mapping between parameter and phenotypic spaces.

The second question is one of fitting method. The most common approach approximates the higher degree polynomial with a linear statistical model, as we have done here. Our results illustrate the limitations of that approach. A more sophisticated, but compute-intensive, approach would be to fit a set of hyperplanes, approximating the surface as a set of n−1-dimensional splines. Another approach is to optimize the fit of the model to the data using one of many nonlinear optimization techniques (Bartholomew-Biggs 2008). Nonlinear optimizations require a good initial guess for the parameter values. We found the values generated by the linear fits were not good guesses, evidently placing the initial point outside the feasible region of the optimization. Further experimentation will be needed to improve the fits using this approach. Finally, one can search systematically for parameter combinations that generate surfaces that match the experimental ones. This is one way of asking how sensitive the phenotypic surfaces are to variations in the parameters’ values. For nonlinear polynomial functions, the relationship between sets of parameter values and generated surfaces will not be regular or easily anticipated: stepwise changes in parameter values will produce “clumps” of generated phenotypes, consistent with the nonlinearity and sloppiness of the system.

We encourage application of our response surface and shape modeling approaches to experiments mixing crop protections or stresses; developing methods to discover producing functions and the various mappings; and detecting and characterizing subspaces that subsume expressed phenotypic points. Methods to more efficiently traverse phenotypic spaces have the potential to accelerate breeding gains.

## Appendix

### List of Supplementary Material

• On FigShare at https://figshare.com/s/3ef69b44d24d0953d625:

1. Supplemental_Material_metadata_readme.txt

2. Supplemental_Data_File_1a.csv

3. Supplemental_Data_File_1b.csv

4. Supplemental_Data_File_2.csv

5. Supplemental_Data_File_3.csv

6. Supplemental_Methods_File_1v2.rtf

7. Supplemental_Figure_S1.png

8. Supplemental_Results_Figure_S2.png

9. Supplemental_Results_File_1.csv

10. Supplemental_Results_Figure_S3.pdf

• On GitHub at https://github.com/tonikazic/univariate_dose_response.git. a public repository:

11. replot MatLab surfaces in R in standard orientation: replot_ann.r

12. generate surfaces according to Eqn 2: modified_eqn.r

13. library of proxies and other helper functions: sweep_fcns.r

14. library of helper functions for analysis: analysis_fcns.r

15. linear estimation of parameters: estimate_exptl_parameters.r

16. standard view for plotting surfaces in 3D: std_view.r
